# PI3Kδ promotes CD4^+^ T-cell interactions with antigen-presenting cells by increasing LFA-1 binding to ICAM-1

**DOI:** 10.1038/icb.2016.1

**Published:** 2016-02-02

**Authors:** Fabien Garçon, Klaus Okkenhaug

**Affiliations:** 1 Laboratory of Lymphocyte Signaling and Development, The Babraham Institute, Babraham Research Campus, Cambridge, UK

## Abstract

Activation of T lymphocytes by peptide/major histocompatibility complex on antigen-presenting cells (APCs) involves dynamic contacts between the two cells, during which T cells undergo marked morphological changes. These interactions are facilitated by integrins. Activation of the T cells increases the binding of the integrin lymphocyte function-associated antigen 1 (LFA-1) expressed by T cells to intercellular adhesion molecule (ICAM)-1 and ICAM-2 expressed by APCs. The signalling pathways that control integrin affinities are incompletely defined. The phosphoinositide 3-kinases (PI3Ks) generate second-messenger signalling molecules that control cell growth, proliferation, differentiation and trafficking. Here we show that in T cells, PI3Kδ attenuates the activation of Rac1, but sustains the activation of Rap1. Consequently, PI3Kδ increases LFA-1-dependent adhesion to form stable conjugates with APCs. Increased Rap1 activity and LFA-1 adhesion were only in part mediated by the downstream kinase Akt, suggesting the involvement of additional phosphatidylinositol(3,4,5)P_3_-binding proteins. These results establish a link between PI3K activity, cytoskeletal changes and integrin binding and help explain the impaired T-cell-dependent immune responses in PI3Kδ-deficient mice.

Phosphoinositide 3-kinases (PI3Ks) catalyse the conversion of phosphatidylinositol(4,5)P_2_ to phosphatidylinositol(3,4,5)P_3_ (PIP_3_). PIP_3_ acts as a lipid second messenger by recruiting PH domain containing proteins to the plasma membrane where they activate signalling pathways that promote proliferation, differentiation, survival and chemotaxis.^1, 2, 3^ The best understood PIP_3_ effector is the serine/threonine kinase Akt, which inactivates Foxo transcription proteins, whereas increases mechanistic target of rapamycin kinase activity.^4, 5^ These pathways are evolutionary conserved and are thought to be responsible for many of the biological functions of PI3Ks. However, it has been estimated that there are up to 50 additional PIP_3_-binding proteins in the human genome and the function of many of these remain to be fully appreciated.^6^ These include numerous guanine exchange factors (GEFs) and GTPase-activating proteins (GAPs) that positively and negatively regulate small GTPases.^7^

Four class I PI3Ks are expressed in mammalian cells. Each consists of a constitutive heterodimer between a p110 catalytic subunit and one of several regulatory subunits. P110α, p110β and p110δ bind to p85α, p55α, 50α, p85β or p55γ (collectively known as p85) to form PI3Kα, PI3Kβ or PI3Kδ, respectively. The p85 regulatory subunits contain SH2 domains that link the p110 subunit to activation by tyrosine kinases. P110γ by contrast binds to a p84 or p101 regulatory subunit and these regulatory subunits are bound by Gβγ subunits released upon engagement of G-protein coupled receptors. We and others have previously demonstrated key roles for PI3Kδ in T cells using kinase-dead p110δ^D910A^ mice, p110δ^−/−^ knockout mice or the small molecule inhibitor IC87114.^2,8, 9^ Inhibition of PI3Kδ in T cells results in a reduction of antigen-induced PIP_3_ accumulation at the immunological synapse; reduced T-cell proliferation; failure of naive T cells to develop into Th1, Th2, Th17 or Tfh subsets; and production of effector cytokines.^10, 11, 12, 13, 14^ PI3Kδ is also required for the expression of certain adhesion and chemokine receptors and in antigen-dependent trafficking of T cells.^15, 16, 17^

Although p110δ^D910A^ T cells showed impaired proliferation when stimulated by peptide antigens *in vitro*, rigorous proliferation of p110δ^D910A^ T cells was achieved using plastic beads coated with high-affinity agonist antibodies.^10, 11, 18^ Similarly, wortmannin blocked antigen stimulated, but not anti-CD3-stimulated T-cell functions.^19^ Because the interactions between T cells and antigen-presenting cells (APC) involve lower affinity interactions than those provided by antibodies bound to beads, we reasoned that PI3Kδ may contribute to increase the stability of the T-cell-APC interaction. Indeed, while monitoring PIP_3_ accumulation at the immune synapse in a previous study we noticed that p110δ^D910A^ and IC87114-treated WT T cells appeared more rounded and tended not to ‘embrace' the APCs as effusively as untreated wild-type (WT) cells did.^12^

T-cell adhesion to APCs is initially mediated by the interaction of T-cell integrins with their ligands expressed on the surface of the APC. Key among the integrins required for the maintenance of the T cell-APC contact is the αLβ2-integrin lymphocyte function-associated antigen 1 (LFA-1) that binds to intercellular adhesion molecule 1 (ICAM)-1 and ICAM-2.^20, 21^ LFA-1 is maintained in a low-affinity conformation on resting T cells, but T-cell receptor (TCR) engagement induces its activation and clustering, which increases adhesion between the T cell and the APC. The importance of these interactions has been highlighted by the finding that deficiency in either LFA-1 or ICAM-1 leads to impaired conjugate formation, reduced responsiveness to antigen, impaired T-cell differentiation and memory T-cell formation.^22, 23, 24, 25^ Rap1 is a small GTPase and a key regulator of LFA-1. Transgenic mice expressing an activating mutant of Rap1A isoform show enhanced adhesion and consequently are hypersensitive to activation.^26^ Therefore, there are similarities between the consequences of modulating LFA-1 activity and PI3K activity in T cells.

Here we show that PI3Kδ activity is required to sustain Rap1 activity after stimulation through the TCR. Consistent with this finding, PI3Kδ is required to increase LFA-1 binding to its ligand ICAM-1 by mechanisms that are partially dependent on Akt and Rap1. In addition, we present evidence for dysregulation of the small GTPase Rac1 and the actin-modifying enzymes cofilin and ERM (ezrin, radixin and moesin). We propose that PI3Kδ coordinates the regulation of cytoskeletal and integrin affinity to establish the formation of stable conjugates between T cells and APCs, which is initiated by low-affinity antigen-dependent interactions.

## Results

### PI3Kδ differentially regulates small G proteins

**Figure 1:**
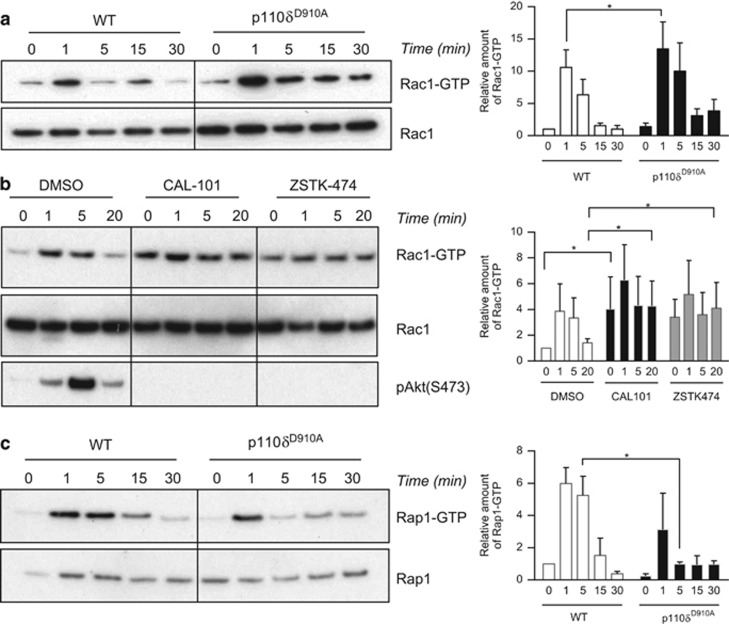
p110δ regulates small GTPases activity in CD4^+^ T cells. The active form of Rac1 (**a**,**b**) and Rap1 (**c**) was purified by GST pull-downs from T cell lysates after stimulation with an anti-CD3 mAb for the indicated times. For quantification, the relative amount of Rac1-GTP or Rap1-GTP was normalised according to the total amount of the respective protein. The data are reported as the fold intensity of the normalised GTP-bound protein observed at *t*=0 in the WT (**a**, **c**) or DMSO (**b**) condition. *P*-values were calculated using a two-way repeated-measures ANOVA. *P<0.05. Data presented are representative of at least three independent experiments.

To investigate the role of PI3Kδ in regulating small GTPase activity, we stimulated WT or p110δ^D910A^ T cells with anti-CD3 and monitored the GTP-bound forms of Rac1, RhoA, Cdc42 and Rap1. RhoA and Cdc42 activity did not change substantially (Supplementary Figures 1a, b), whereas Rac activation occurred rapidly after stimulation with anti-CD3 (Figure 1a). Contrary to our expectations, Rac1 showed a tendency to be more strongly activated and this activity was maintained longer in p110δ^D910A^ T cells than in WT T cells (Figure 1a). Acute inhibition of PI3K by using either the PI3Kδ-specific inhibitor CAL-101 or a pan-PI3K inhibitor (ZSTK-474) led to enhanced basal Rac1 activity and CAL-101-treated samples, similar to the p110δ^D910A^ mutation, maintained higher Rac activity (Figure 1b). By contrast, the activation of Rap1 was attenuated in p110δ^D910A^ T cells relative to WT T cells, particularly at later time points (Figure 1c). Thus, sustained Rap1 activation requires PI3Kδ activity.

### Reduced cofilin inactivation and morphological changes in PIK3δ-deficient T cells

**Figure 2:**
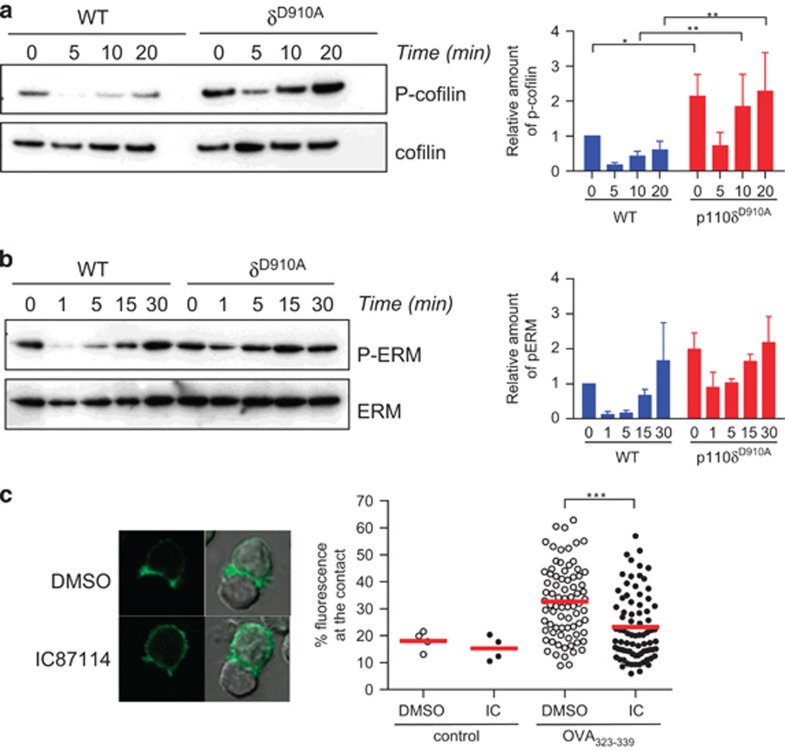
PI3Kδ controls cofilin and ERM regulation and cell polarisation. WT and p110δ^D910A^ CD4^+^ T cells were activated with anti-CD3 and anti-CD28 mAbs for the indicated times and lysed. Lysates were then analysed with the indicated antibodies in order to measure cofilin activity (**a**) or ERM phosphorylation (**b**). For quantification, relative amounts of phospho-cofilin or phospho-ERM were normalised to the total amount of cofilin or ERM. *P*-values were calculated using a two-way repeated-measures ANOVA. *P<0.05. Data presented are representative of three independent experiments. (**c**) Actin-GFP expressing OT2 CD4^+^ T cells were pre-treated with DMSO or the p110δ specific inhibitor IC87114 before being incubated with B cells loaded with OVA_323-339_ peptide. After 30 min, cells were fixed and the intensity of the fluorescence of actin-GFP at the contact zone measured. Data are representative of at least three independent experiments.

Cofilin is an actin-binding protein important for actin reorganisation. In resting, T-cell cofilin is maintained inactive by phosphorylation on Serine 3 by serine/threonine kinases LIMK1.^27^ LIMK1 is activated through Rac small GTPase and its effector PAK. Cofilin is dephosphorylated and activated by Slingshot phosphatases in a PI3K-dependent manner.^28^ Immunoblot analysis with an antibody specific for P-Serine 3 showed that activation of WT T cells decreased phosphorylation of cofilin in a time-dependent manner (Figure 2a). By contrast, p110δ^D910A^ T cells had higher basal level of cofilin phosphorylation, which was incompletely dephosphorylated upon T-cell stimulation (Figure 2a). These results are consistent with higher Rac activity in p110δ^D910A^, which may lead to increased LIMK activity, shifting the balance towards more phosphorylation of cofilin.

We also examined the role of p110δ in the phosphorylation of the ERM proteins that link actin filaments to proteins in the plasma membrane. The ERM proteins have been shown to be important for cell deformability and immunological synapse formation and are also regulated by Rac.^29^ Similar to cofilin, ERM-dephosphorylation was less efficiently induced in p110δ^D910A^ T cells than in WT T cells (Figure 2b). These results suggest that PI3Kδ inhibition uncouples the TCR from key proteins involved in actin cytoskeleton rearrangements, possibly by altering the flux through the GDP- and GTP- bound states of Rac.

We next examined the effect of inhibiting PI3Kδ on actin reorganisation and cell shape changes during T-cell activation. Upon encountering an antigen-bearing APC, T cells reorganise the actin cytoskeleton and polarise towards the contact area. In order to follow this event we transfected OT-II CD4^+^ T cells with actin-GFP and allowed them to interact with B cells loaded with OVA_323-339_ peptide before imaging them. Pre-treatment of the T cell with a p110δ-specific inhibitor induced a reduction of the actin-GFP signal at the contact area (Figure 2c) and quantitative analysis of the intensity of fluorescence at the contact confirmed that PI3Kδ inhibition suppressed dynamic actin reorganisation required for productive interactions with APCs.

### PI3Kδ is required for optimal LFA-1-dependent adhesion and T-cell spreading

**Figure 3:**
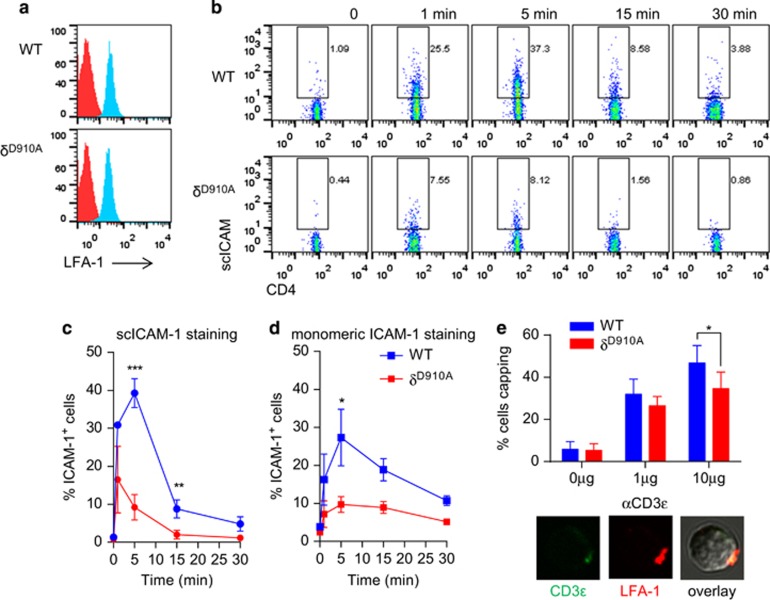
Impaired TCR-induced LFA-1 activation in p110δ^D910A^ T cells. (**a**) Expression level of LFA-1 on WT and p110δ^D910A^ CD4^+^ T cell is shown. (**b**, **c**) CD4^+^ T cells were incubated with sc-ICAM-1 and stimulated with anti-CD3ɛ. After the indicated times, the reaction was stopped with 4% PFA. Bound scICMA-1 was detected by flow cytometry. (**b**) Dot plots of a representative experiment are depicted. The percentage of positive cells for ICAM-1 is shown (**c**). Data show means of three independent experiments. (**d**) Reduced LFA-1 affinity in p110δ^D910A^ T cells. CD4^+^ T cells were incubated with monomeric ICAM-1 Fc and stimulated with anti-CD3ɛ. Bound ICAM-1 Fc was detected by staining with Fc-specific PE-labelled F(ab')_2_ fragment. Data show means of three independent experiments. (**e**) p110δ^D910A^ T cells show normal LFA-1 capping. Cells were stimulated with anti-CD3 and LFA-1 was visualised with a PE-labelled anti-CD11a. Histogram show percentage of T cells presenting a LFA-1 cap. Pictures show examples of LFA-1 distribution on activated T cells.

The reduced Rap1 activity observed in p110δ^D910A^ T cells suggested that PI3Kδ could be important for LFA-1 binding to ICAM-1. WT and p110δ^D910A^ T cells showed the same level of LFA-1 expression (Figure 3a). Integrin activation occurs through two distinct mechanisms, namely an increase of affinity and/or avidity.^20^ To examine the effect of the absence of PI3Kδ activity on LAF-1 affinity and avidity we used a ligand-complex-based-adhesion-assay.^30^ In this assay, p110δ^D910A^ T cells showed reduced binding to soluble ICAM-1 clusters upon TCR stimulation (Figures 3b and c). By contrast, p110δ^D910A^ T cells activated with CCL19 showed normal binding to ICAM-1 (Supplementary Figure 2A), consistent with previous results.^31^ Similarly, addition of PMA or Mg increased ICAM-1 binding to p110δ^D910A^ T cells (Supplementary Figure 2B). These results demonstrate that LFA-1 can be activated by other pathways in p110δ^D910A^ T cells and suggest a specific role for PI3Kδ in linking the TCR to LFA-1 activation. LFA-1 binding was further analysed by measuring the ability of cells to bind monomeric ICAM-1. As with clustered ICAM-1, p110δ^D910A^ T cells failed to bind to monomeric ICAM-1 (Figure 3d). A similar result was obtained by treating WT T cells with the PI3Kδ-specific inhibitor CAL-101 before measuring binding to monomeric ICAM-1 (Supplementary Figure 2c). However, analysis by confocal microscopy revealed that LFA-1 capped normally in activated p110δ^D910A^ T cells, except at the highest concentration of anti-CD3 (Figure 3e). These results therefore suggest that PI3Kδ regulates LFA-1 primarily by increasing its affinity for ICAM-1, although we cannot exclude a role for PI3Kδ in regulating LFA-1 microclusters.

**Figure 4:**
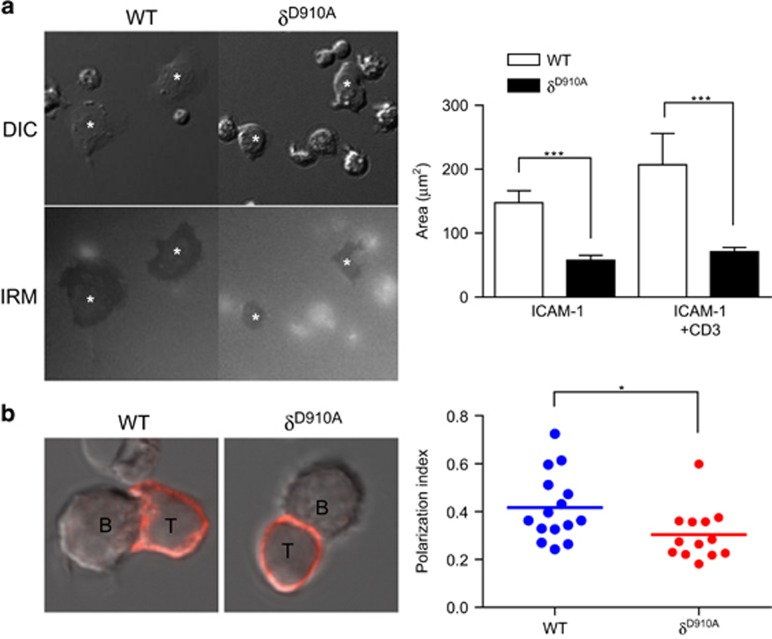
p110δ is required for T-cell spreading on ICAM-1 and APC. (**a**) WT and p110δ^D910A^ CD4^+^ T-cell blasts were settled on anti-CD3 mAb plus ICAM-1coated coverslip. DIC and IRM images are shown and quantified. Data in the histogram represent the means of two independent experiments. (**b**) p110δ is required for T-cell spreading on APC. The polarisation index of WT or p110δ^D910A^ CD4^+^ T-cell blasts interacting with OVA_323-339_-loaded APC was quantified as described in Methods. A round cell would have a value of 0. Representative images of a conjugate formed by WT or p110δ^D910A^ T cells (labelled with a PE-anti-CD3 mAb) are shown. Data are representative of three independent experiments.

In order to form and maintain a viable cell–cell contact with their APCs, T cells need to spread and extend membrane tethers to maximise the contact area with the APC. To mimic this effect, we measured the spreading of T cells that had been deposited on coverslips coated with ICAM-1 alone or ICAM-1 and anti-CD3. T-cell spreading was enhanced by the presence of anti-CD3 as indicated by their increased measured contact area (Figure 4a). However, p110δ^D910A^ T cells spread less extensively than WT T cells (Figure 4a). Next, OT2 TCR-transgenic CD4^+^ T cells were incubated with APC-presenting OVA_323-339_ peptide and T cells making cell–cell contacts were analysed. We found that p110δ^D910A^ T cells polarised less extensively when they interacted with APCs (Figure 4b).

**Figure 5:**
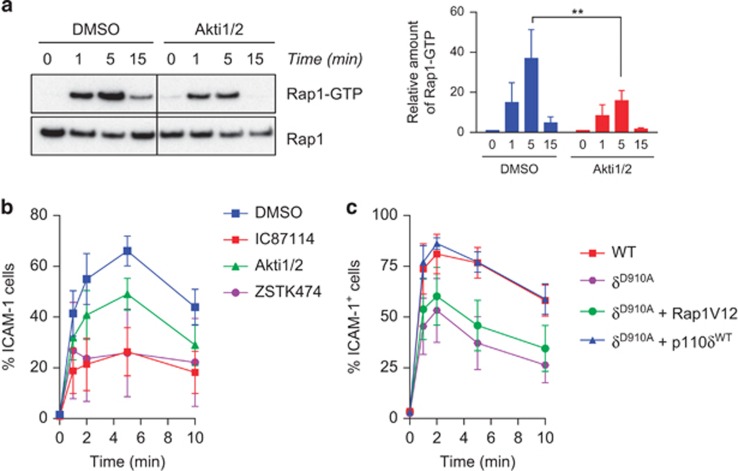
Inhibition of Akt reduces Rap1 and LFA-1 activation. (**a**) The active form of Rap1 was pull-down from CD4^+^ T cells pre-treated by DMSO or the Akt inhibitor Akti1/2. Quantification of the signal was done as in Figure 1. (**b**) DMSO or Akti-treated T cells were stimulated with anti-CD3 mAb in presence of scICAM-1 for the indicate times. Bound APC-labelled scICAM-1 on surface of CD4^+^ T cells was detected by flow cytometry. (**c**) WT and p110δ^D910A^ T cells transfected with constitutively active Rap1 or WT p110δ were assessed for LFA-1 binding to scICAM-1. (**a–c**) All data are representative of at least three independent experiments.

As Akt is one of the main effectors of PI3K we wanted to assess its contribution to PI3Kδ-dependent inside–out signal to LFA-1. T cells treated with an inhibitor of Akt showed reduced activation of Rap1 upon TCR engagement (Figure 5a). This was accompanied by a reduced binding of LFA-1 to ICAM-1 (Figure 5b). However, this reduction was not as strong as the one observed when T cells were treated with the PI3Kδ inhibitor IC87114 or the pan-PI3K inhibitor ZSTK-474 (Figure 5b), suggesting that another PI3Kδ-dependent pathway is also involved. We also asked whether we could restore binding to ICAM-1 by transducing cells with an activated form or Rap1 (Rap1^V12^) in p110δ^D910A^ T cells. We observed a partial rescue of the binding of LFA-1 to ICAM-1 in cells expressing Rap1^V12^ (Figure 5c). However the binding enhancement never reached the level observed for WT T cells or p110δ^D910A^ T cells transduced with WT p110δ cDNA (Figure 5c). This suggests that Rap1-independent pathways can also contribute to LFA-1 activation downstream of PI3Kδ in T cells.

### T cell-APC cell conjugates formed by p110δ^D910A^ CD4^+^ T cells are unstable

**Figure 6:**
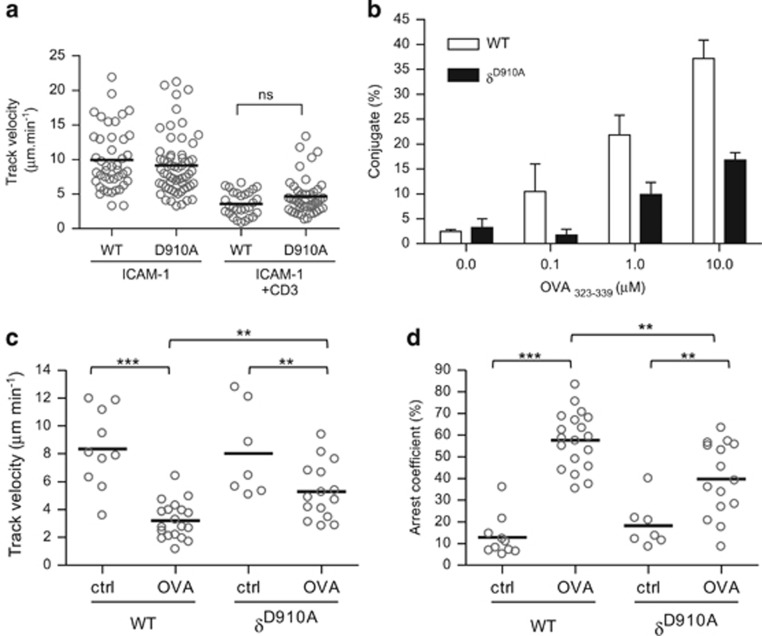
Inhibition of p110δ disrupts T cell-APC conjugate formation. (**a**) WT and p110δ^D910A^ CD4^+^ T-cell blasts were loaded onto surfaces coated with ICAM-1 alone or with ICAM-1 and anti-CD3 and imaged by video microscopy for 10 to 15 min. Cell movements were analysed by Volocity software. Data are representative of three independent experiments. (**b**) WT and p110δ^D910A^ OT2 CD4^+^ T-cell blasts were incubated with OVA_323-339_-loaded APC for 30 min and assessed for conjugate formation as described in Methods. (**c**–**d**) p110δ^D910A^ T cells do not slow down as much as the WT when presented antigen by the APC. Data show means of the velocity (**c**) and arrest coefficient (**d**) of WT and p110δ^D910A^ OT2 CD4^+^ T-cell blasts during their interaction with APC in presence or absence of OVA_323-339_ peptide. Data are the mean values of three independent experiments.

Within lymph nodes, T cells move about at relatively high rates until they interact with APCs presenting cognate peptide antigens, which causes them to arrest, thus enabling the establishment of further contacts between the T cell and the APC.^32, 33^ To measure the ability of the TCR to send a signal to arrest p110δ^D910A^ T cells, we plated T cells on ICAM-coated slides as previously described.^34^ In the absence of stimulation, p110δ^D910A^ T cells migrated at a similar velocity as WT T cells and stimulation with anti-CD3 reduced the motility of WT and p110δ^D910A^ T cells with similar efficiency (Figure 6a). These results indicate that the TCR-dependent ‘stop' signal is intact in p110δ^D910A^ T cells under these conditions. To determine whether PI3Kδ is required for sustained conjugate formation between T cells and APCs, we incubated WT or p110δ^D910A^ OT2 CD4^+^ T cells with APCs loaded with increased concentration of OVA_323-339_ peptide and counted the number of conjugates formed. WT OT2 T cells formed stable conjugates with APCs loaded with OVA_323-339_ peptide in a dose-dependent manner (Figure 6b). However, at each peptide concentration, p110δ^D910A^ T cells formed fewer conjugates (Figure 6b). When WT or p110δ^D910A^ OT2 T cells were incubated with APCs not loaded with peptides, they moved at similar speeds (8.3±0.87 μm min^−1^ for WT and 8±1.2 μm min^−1^ for p110δ^D910A^) suggesting that p110δ was not involved in the control of the basal motility of the T cells (Figure 6c). However, when the APCs were presenting OVA_323-339_ peptide, WT OT2 T cells migrated with a lower mean velocity (3.2±0.3 μm min^−1^) and were more likely to reduce their movement (57.6±3% moved at <2 μm min^−1^) (Figures 6c and d). By contrast, p110δ^D910A^ T cells retained a significantly higher mean velocity (5.3±0.5 μm min^−1^), and were less likely to arrest their movement (39.8±4.4%) in presence of peptide antigen. These results suggest that PI3Kδ contributes to formation of stable interactions between T cells and APC-presenting cognate peptides antigens.

### p110δ^D910A^ CD4^+^ T cells form less-stable T cell-APC interactions in lymph node slices

**Figure 7:**
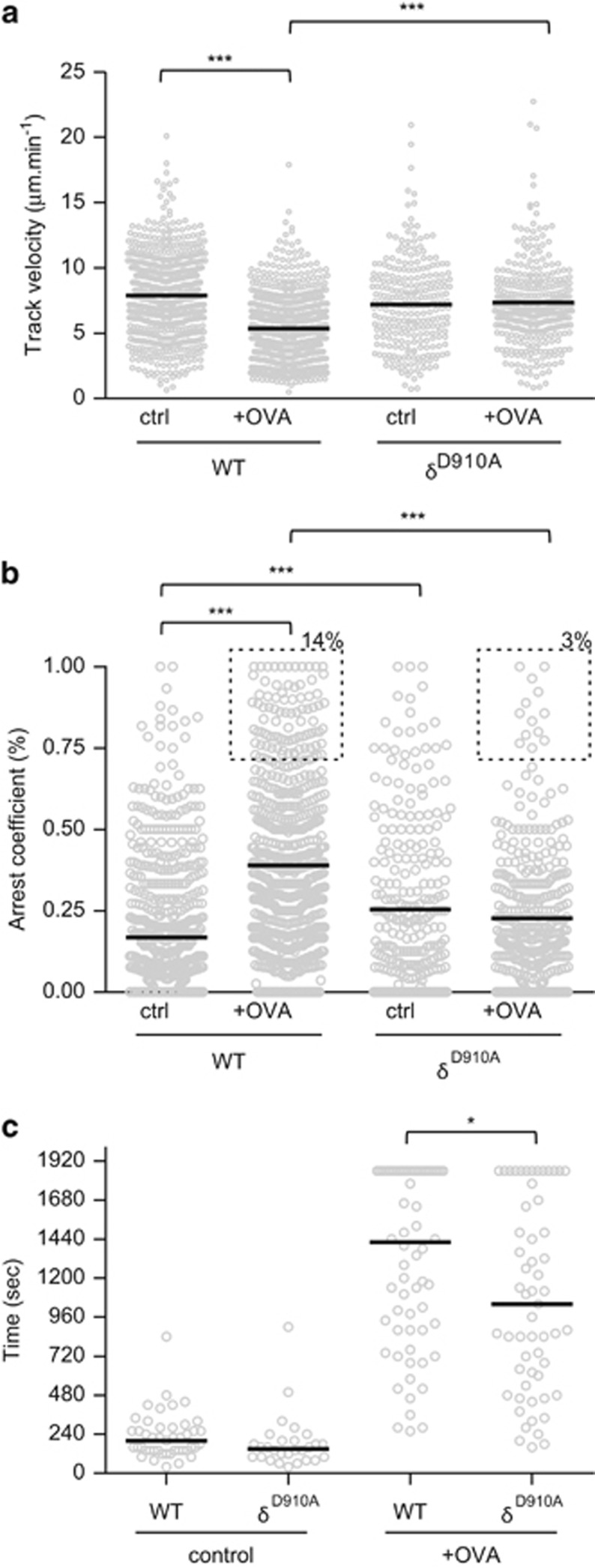
PI3Kδ is important for T-DC interactions in lymph node slices. CD4^+^ T-cell blasts labelled with CMFDA and RFP^+^ DCs were added to congenic lymph node slice in the presence or absence of OVA_323-339_ peptide. Mean velocity (**a**) and arrest coefficient (**b**) of WT and p110δ^D910A^ OT2 cells in presence or absence of peptide. Dashed boxed in (**b**) indicate the frequency of WT and p110δ^D910A^ OT2 cells with an arrest coefficient >0.7 (arrested). (**c**) Contact times between OT2 CD4^+^ T cells and DCs. (**a–c**) Data are representative of at least two independent experiments.

Our *in vitro* results indicated that p110δ^D910A^ T cells form less-stable conjugate using lipopolysaccharide-primed B cells as APCs. In the lymph node, T cells move in three dimensions along a fibroreticular network where dendritic cells (DCs) act as the main type of APC during the initiation of immune responses.^35^ We therefore investigated whether the effects of PI3Kδ-deficiency *in vitro* were also observed when DCs present peptide antigen within the context of the lymph node microenvironment. To this end, we prepared agarose-embedded lymph node slices, which previously have been shown to support normal lymphocyte motility.^36^ When added to lymph node slices together with DCs not presenting OVA_323-339_ peptide, both WT and p110δ^D910A^ OT2 CD4^+^ T cells moved at similar mean velocities (7.9±0.1 μm min^−1^ and 7.2±0.2 μm min^−1^, respectively) (Figure 7a). When the cells were added to a slice together with DCs presenting OVA_323-339_ peptide, the WT OT2 T cells moved at a reduced velocity (5.3±0.1 μm min^−1^), whereas the p110δ^D910A^ OT2 T cells did not significantly reduce their velocity (7.3±0.19 μm min^−1^). The reduced ability to form stable conjugate of the p110δ^D910A^ OT2 T cells was further indicated by their failure to increase their arrest coefficients in lymph node slices containing OVA_323-339_ peptide (Figure 7b). The median interaction times between T cells and antigen-bearing DCs in lymph node sections were also reduced when p110δ^D910A^ where added to the slices (Figure 7c). These data show that PI3Kδ is required for the establishment of sustained contacts with DCs in response to antigenic challenge in a lymph node. Future experiments will establish whether p110δ^D910A^ cells also fail to maintain stable interactions in the context of an inflamed lymph node.

## Discussion

In the study we have investigated the effect of inhibiting PI3Kδ on the ability of CD4^+^ T cells to form productive conjugates with APCs.

To our initial surprise, we found Rac activity to be intact, or even enhanced, in p110δ^D910A^ T cells after stimulation with anti-CD3. This contrasts to observations in neutrophils where PI3K has been shown to positively regulate Rac activity via the protein P-Rex1, which catalyses the exchange of GDP for GTP on Rac using its GEF domain.^37^ However, in subsequent studies, no effect of P-Rex1 deficiency on lymphocyte function was found^38^ (our unpublished data). Vav1 is a key regulator of Rac activity in T cells and its capacity to regulate Rac is dependent on the ability of the Vav-PH domain to bind PtdIns(4,5)P_2_ or PIP_3_.^39^ However, as the Vav1 PH domain does not discriminate effectively between PIP_3_ and its much more abundant precursor PtdIns(4,5)P_2,_ it is unlikely that Vav1 is acutely regulated by PI3Ks. The Rac GEF Dock2 has also been considered to bridge PI3K signalling to Rac activation. Rac activity is reduced in Dock2^−/−^ T cells stimulated via the TCR or through chemokines.^40, 41, 42^ During the activation of CD8^+^ T cells, actin is cleared from the immune synapse to facilitate the delivery of cytotoxic granules.^41, 43^ Le Floc'h and colleagues noted reduced cell spreading and depletion of actin at the centre of the immune synapse in CD8^+^ T cells, lacking Dock2 or that had been inhibited with IC87114.^41^ Our data are consistent with this study with regards to cell spreading and impaired actin reorganisation, but we cannot conclude that this is simply a consequence of impaired Rac activation. Inactivation of Rac is mediated by GAP, which stimulates the intrinsic hydrolysis activity of small GTPases. ArhGAP15 is regulated in a PIP_3_-dependent manner and ArhGAP15^−/−^ neutrophils and macrophages show enhanced Rac activity, which is antagonised by PI3K signalling.^44, 45^ Although TCR-dependent Rac activity in ArhGAP15^−/−^ T cells is unperturbed (Garçon F, Costa C, Hirsch E, Okkenhaug K, manuscript in preparation), these results indicate that Rho-family GAPs as well as Rho-family GEFs need to be taken into account when considering the effect of PI3K inhibition on Rac activity in different cell types and downstream of distinct receptors. Indeed, during phagocytosis, the activation of up to three different PI3K-dependent Rac-GAPs is required for actin clearance in a manner reminiscent of that observed during CTL activation by antigen.^46^ We propose that impaired actin reorganisation in p110δ^D910A^ CD4^+^ T cells is a consequence of altered dynamics in the cycling between the GTP- and GDP-bound states of Rac, which can be affected both by PIP_3_-dependent GEFs and GAPs.

Rap1 activation was reduced in p110δ^D910A^ T cells stimulated with anti-CD3, and binding of ICAM-1 to LFA-1 was also impaired suggesting that PI3Kδ could regulate LFA-1 activation through Rap1. The capacity of LFA-1 to interact with its ligand ICAM-1 is regulated by two processes: conformational change, leading to higher affinity binding, and clustering, leading to increased avidity.^20^ Although activated Rap1 has been reported to regulate both the avidity and the affinity of LFA-1, a constitutively active mutant of Rap1 preferentially increased LFA-1 avidity in primary T cells.^26, 47^ Our results suggest that PI3Kδ regulates the affinity rather than the avidity of LFA-1, as PI3Kδ inhibition suppressed binding of LFA-1 on T cells to a soluble ligand without affecting capping of LFA-1 on the T cells. If the main effect of activated Rap1 is to increase LFA-1 clustering, then this would explain the failure of a constitutively active form of Rap1 (Rap1V12) to rescue the binding to ICAM-1 by LFA-1 expressed by p110δ^D910A^ T cells. This may also explain why in a previous study we found that p110δ^D910A^ T cells bound normally to ICAM-1 plate-based adhesion assay.^10^ The plate-based adhesion assay used in that study cannot discriminate between affinity- and avidity-mediated changes in adhesiveness. The present study shows that although p110δ^D910A^ T cells were able to cap LFA-1 at the surface and bind plate-bound ICAM-1, they failed to spread and had a very small surface of contact.

Contrary to our initial hypothesis, the reduction of Rap1 activity we observed when PI3Kδ was inhibited is not in itself sufficient to explain the reduced binding of LFA-1 to ICAM-1. In addition to regulating integrin affinity, Rap1 is also required for cell spreading and actin dynamics.^48^ Rap1 does so by promoting the dephosphorylation and activation of the actin-severing protein cofilin. Cofilin phosphorylation is also increased via Rac upon TCR/CD28 engagement.^27^ Our data show that PI3Kδ inhibition suppressed cofilin dephosphorylation and dynamic actin reorganisation required for cell shape modification and productive interactions with APCs, which may be another consequence of reduced Rap1 activity. How the reduced Rap1 activation and increased Rac1 activity in p110δ^D910A^ T cells is integrated to regulate cytoskeletal rearrangements and integrin activation still remains to be fully elucidated, however.

Interestingly, Rap1 activity was also reduced when Akt was inhibited. Inhibition of Akt also led to a small decreased binding to ICAM-1 but not as strong as that observed in p110δ^D910A^ T cells or T cells treated with the PI3Kδ inhibitor IC87114. These results implicate Akt in the regulation of integrins, but also suggest that PI3K effectors other than Akt mediate PI3K-dependent integrin activation. Which other PIP_3_-binding proteins could contribute to Rap1 activation and increased LFA-1 affinity? SKAP1 is composed of a unique NH_2_-terminal region followed by a PH and a SH3 domain.^49, 50^ Upon TCR engagement, SKAP1 is recruited to an ADAP-SLP-76 complex via the interaction of its SH3 domain with a proline-rich region in ADAP and then interacts with RapL and allows its recruitment to Rap1 and LFA-1.^50^ Like p110δ^D910A^ mice, SKAP1-deficient mice are impaired in forming T-cell–APC conjugates *in vitro* and *in vivo.*^50, 51^ A mutation R131M in the PH domain, which is predicted to uncouple SKAP1 from regulation by PIP_3_ inhibits the ability of SKAP1 to enhance LFA-1 activation in a cell line.^49, 52^ Kindlin-3, a key LFA-1 co-activator deleted in leukocyte-adhesion deficiency-III, also possesses a PIP3-specific PH domain in its FERM domain F2.^53^ Kindlin-3 is required for induction of the high-affinity conformation of LFA-1.^54^ Moreover, the PH domain of Kindlin-3 is essential for LFA-1-mediated regulation of lymphocyte adhesion and migration.^55^ The intermediate affinity conformation of LFA-1 requires Talin-1, whereas further conformational changes associated with the high-affinity state are Kindlin-3 dependent.^54^ Further work will help delineate how different PIP_3_-binding proteins co-ordinate the regulation of LFA-1 affinity and avidity. These studies may be facilitated using human T cells as antibodies that distinguish between low-, medium- and high-affinity forms of human LFA-1 are available.

T-cell activation within the spleen or lymph nodes occurs through multiple stages over a period of up to 30 h, starting with transient contacts that allow T cells to scan a large number of APCs and then proceeding to longer-lasting interactions once an APC-bearing cognate peptide antigen is encountered.^33, 56, 57^ Although shorter interactions can stimulate T-cell proliferation and cytokine production, sustained dynamic interactions are thought to be required to initiate differentiation programs and for immunological memory.^21, 22^ Consistent with previous results,^36, 58^ we found that PI3K inhibition did not affect the basal motility of T cells moving through lymph nodes in absence of antigen. However, when presented with antigen, p110δ^D910A^ T cells failed to establish stable conjugates with APCs. This defect is likely to be a contributing factor to the impaired T-cell responses to antigen in p110δ^D910A^ mice.

## Methods

### Mice

OT2 and p110δ^D910A^ mice on the C57BL/6 genetic background were described previously (and references therein).^12^ Mice expressing tdRFP from the Rosa26 locus were obtained from the European Mouse Mutant Archive.^59^ Mice were maintained under specific pathogen-free conditions. All protocols involving live animals were approved by the UK Home Office and the Babraham Institute Animal Welfare, Experimental Ethical Review Committee and in accordance with the Animals (Scientific Procedures) Act 1986.

### Reagents

OVA_323-339_ peptide was synthesised by Southampton Polypeptides. IC87114 was synthesised as described previously.^14, 60^ Akt inhibitor VIII (Akti ½) was purchased from Merck Millipore (Billerica, MA, USA) and used at 2 μm. ZSTK-474 was purchased from Selleck (Houston, TX, USA) and used at 100 nM. The different glutathione S-transferase (GST) fusion proteins containing the CRIB (Cdc42/Rac1 interactive binding) domain of PAK (GST-PAK-CRIB), the Rho-binding domain (RBD) of Rhotekin (GST-Rhotekin-RBD) or the Ras-binding domain (RBD) of RalGDS (GST-Ral-RBD) were a gift from Heidi Welch (Babraham Institute). HA-Rap1V12 was cloned into MIGR-1-IRES-GFP using the Gateway cloning technology from Life technologies (Grand Island, NY, USA). The human p110δ retroviral construct was described previously.^61^ The recombinant murine MIP-3β (CCL19) was obtained from Peprotech (London, UK).

### Antibodies

The following antibodies were used in this study: anti-Rac1 (clone 23A8) from Millipore, anti-Cdc42, anti-RhoA and anti-Ezrin from Santa Cruz Biotechnology (Santa Cruz, CA, USA), anti-Rap1 antibody from Epitomics (Burlingame, CA, USA) and Cell Signaling Technology (Boston, MA, USA), anti-cofilin, anti-phospho-cofilin (Ser3) and anti-phospho-ERM from Cell Signaling Technology, anti- mouse CD11a (clone M17/4) and anti-CD4 from eBioscience (San Diego, CA, USA).

### Cell purification and transfections

Naive T cells were purified from lymph nodes by magnetic sorting (Miltenyi Biotec, Auburn, CA, USA). For total CD4^+^ T-cell purification, non-CD4^+^ T cells were labelled with fluorescein isothiocyanate-conjugated anti-CD8, anti-CD69, anti-CD25, anti-B220, anti-CD49b and anti-major histocompatibility complex class II, followed by negative selection with anti-fluorescein isothiocyanate magnetic cell sorting beads (Miltenyi Biotec). More than 95% of the cells isolated were CD4^+^.

OT2 T-cell blasts were obtained by stimulation of cells from spleens with 1 μm OVA_323-339_ peptide for 6 days. CD4^+^ T cells were purified by magnetic sorting and cultured with 20 ng ml^−1^ of interleukin-2. Before any experiments, cells were washed to remove interleukin-2 and rested for 2 h. For the actin-GFP analysis, OT2 T-cell blasts were transfected with 6 μg of pcDNA3-actin-GFP with the Amaxa Nucleofactor technology (Lonza, Basel, Switzerland) according to the manufacturer's instructions.

APCs used in this study were T-cell depleted splenocytes isolated with anti-mouse Thy1.2 (Sigma-Aldrich, St Louis, MO, USA) and rabbit complement (Cedarlane, Burlington, ON, USA), followed by purification of viable cells using Lympholyte-M (Cedarlane). The APCs were activated by overnight exposure to lipopolysaccharide with or without the OVA_323-339_ peptide.

DCs were purified from collagenase-digested spleen and isolated by magnetic sorting using CD11c microbeads (Miltenyi Biotec).

### GST pull-downs and western blot analysis

Purified CD4^+^ T cells (2–5 × 10^6^) were incubated with anti-CD3 (2C11 at 10 μg ml^−1^) alone or in combination with anti-CD28 (37.51 at 10 μg ml^−1^) on ice for 30 min. Cells were washed once in ice-cold RPMI, incubated 2 min at 37 °C and then stimulated with goat anti-hamster immunoglobulin G (Jackson ImmunoResearch Laboratory, West Grove, PA, USA) for the indicated times. Cells were lysed in ice-cold lysis buffer (50 mM 4-(2-hydroxyethyl)-1-piperazineethanesulfonic acid, 150 mM NaCl, 10 mM NaF, 10 mM iodoacetamide, 1% NP-40, 1 mM phenylmethylsulfonyl fluoride and protease inhibitors) and mechanically sheared using a needle and syringe. Proteins were resolved on NuPage 4–12% BisTris gels (Invitrogen, Life Technologies Ltd., Paisley, UK), transferred to polyvinylidene difluoride membranes and probed with the indicated antibodies.

For small G proteins analysis, lysates were incubated with the appropriate GST fusion protein (GST-PAK-CRIB, GST-Rhotekin-RBD and GST-Ral-RBD), coupled to glutathione-Sepharose beads for 2 h at 4 °C. Pull-downs were washed three times in ice-cold lysis buffer, and resolved as described above.

### Imaging of T-cell conjugates and migration on slides

CD4^+^ T-cell–APC conjugate formation and imaging were performed as described previously.^12^

To examine T-cell motility *in vitro*, GFP-positive CD4^+^ T cells were added to the APCs already adhered to a coverslip and images were taken every 15 s. For velocity measurement, GFP^+^ cells were tracked to draw a path using the Imaris software.

To measure the number of conjugates formed, for each coverslip three different fields of view randomly chosen were imaged with a × 40 objective. In order to differentiate the T cells we used a PE-anti-CD3ɛ (2C11) antibody. T cells forming a close contact with an APC were considered positive for conjugate formation.

Actin redistribution at the T-cell-APC interface was defined as the ratio of the intensity of fluorescence at the contact and the intensity of fluorescence of the rest of the membrane. Background noise was determined as the average signal in an area without cell and was subtracted from the overall output. Images of conjugates were obtained on an Olympus FV-1000 system consisting of an Olympus IX81 microscope fitted with an Olympus Plan super Apochromat × 60/1.4 numerical aperture oil objective (Olympus Europa GmbH, Hamburg, Germany).

### ICAM-1 complexes-based adhesion assays

To generate ICAM-1-Fc-F(ab')_2_ complexes (scICAM-1) APC- or phycoerythrin-labelled anti-human Fcγ-specific immunoglobulin G F(ab')_2_ fragments (Jackson ImmunoResearch) and recombinant mouse ICAM-1 Fc (R&D Systems, Minneapolis, MN, USA) were incubated at 4 °C for 30 min in phosphate-buffered saline (PBS) as described previously.^30^

Primary CD4^+^ T cells were incubated for 30 min at 4 °C with 1 μg ml^−1^ of anti-CD3ɛ (2C11). The cells were then activated by cross-linking with anti-Armenian Hamster immunoglobulin G at 37 °C in presence of scICAM-1. For the control experiments, the cells were activated with PMA (51 ng ml^−1^) or Mg^2+^/EGTA (10 mM/1 mM) or CCL19 (250 ng ml^−1^). After the indicated time the reaction was stopped by fixation with 4% paraformaldehyde for 5 min. The samples were then washed in PBS+10% fetal calf serum, stained with phycoerythrin- or APC-labelled anti-CD4 and analysed on a fluorescence-activated cell sorting-Calibur.

For LFA-1 clustering experiments, T cells were incubated with anti-CD3ɛ for 30 min on ice. Cells were then washed and cross-linked with anti-Armenian Hamster immunoglobulin G for 30 min at 37 °C. Cells were fixed with 4% paraformaldehyde, washed and blocked with 10% fetal calf serum-PBS for 30 min. Cells were then stained for anti-CD11a and imaged on a Olympus FV-1000 microscope fitted with a Olympus Plan super Apochromat × 60/1.4 numerical aperture oil objective.

### Retroviral transduction of T-cell blasts

WT and p110δ^D910A^ T-cell blasts were obtained by stimulating whole-lymph node cell suspension with anti-CD3 mAb (2C11 clone at 1 μg ml^−1^) and recombinant human interleukin-2 (20 ng ml^−1^) for 2 days. 24 h before the transduction, six-well non-tissue culture plates were coated with Retronectin (Takara Bio Inc., Otsu, Japan) at 50 μg ml^−1^ in PBS overnight at 4 °C. After removing the retronectin, the plates were blocked in PBS+0.5% bovine serum albumin at room temperature for 30 min. The virus-containing supernatant from Plat-E packaging cells was added to the plate and centrifuged at 2000 g at 32 °C for 90 min. After discarding the supernatant the same procedure was repeated with a second aliquot. T-cell blasts (2 × 10^6^) in culture media with interleukin-2 were added to the virus-coated plate and centrifuged at 500 g for 30 min. Cells were used 2 days after the transduction.

### Lymph node slice imaging

Lymph nodes preparation was mostly done as described previously.^36^ For antigen-dependent T-cell motility, slices were loaded with 10 μm of OVA_323-339_ peptide and incubated for 1 h at 37 °C. For T cell-DC interactions, 0.2 × 10^6^ RFP^+^ DCs loaded with 10 μm of OVA_323-339_ peptide were incubated on the slice for 30 min prior to the addition of the T cells. 0.2 × 10^6^ OT2 CD4^+^ T cells, labelled with 1 μm of the cell tracker CMFDA, were then incubated on the slice for 30–60 min in the incubator. The preparation was then thoroughly washed and placed in the perfusion chamber on the microscope. Image were acquired every 20 s for 10–30 min with a × 10 objective on an Olympus CellR system or with a × 20 water immersion objective on a Zeiss 7MP two-photon system. Raw images were treated with Imaris software (Bitplane, Zürich, Switzerland). Imaris Spots module was used to track objects and calculate cell coordinates over time. The resulting tracks were manually checked. Contact times between T cells and DCs, defined as a close association persisting for >1 min, were scored manually by counting the number of frames they persisted for.

### Quantification of the normalised compactness

The normalised compactness (Cn) or polarisation index, a measure of the shape of the cell, was determined as previously described.^62^ The contours of the T cell were determined using the differential interference contrast (DIC) or interference reflection microscopy (IRM) image. From the contours we estimated the shape of the cell according to the formula Cn=1−(4*πA*/*P*^2^) where *A* is the area and *P* the perimeter. A value of 0 represents a perfect circle (non-polarised cell) and a value of 1 a straight line (fully polarised cell).

### Data and statistical analysis

Images were assembled and analysed with Imaris Bitplane and the Volocity software package (Improvision, Waltham, MA, USA). All statistical analyses were performed using Prism 6.0 software (GraphPad Software, San Diego, CA, USA).
