# Supplementary material for: PI3Kδ promotes CD4+ T-cell interactions with antigen-presenting cells by increasing LFA-1 binding to ICAM-1
Source: Immunol Cell Biol. 2016 Feb 2;94(5):486–95. doi: 10.1038/icb.2016.1 (PMC4829101; doi:10.1038/icb.2016.1)
Supplement: Supplementary Figures [file icb20161x1.pdf]

Supplemental Figure 1

(to accompany main Figure 1)

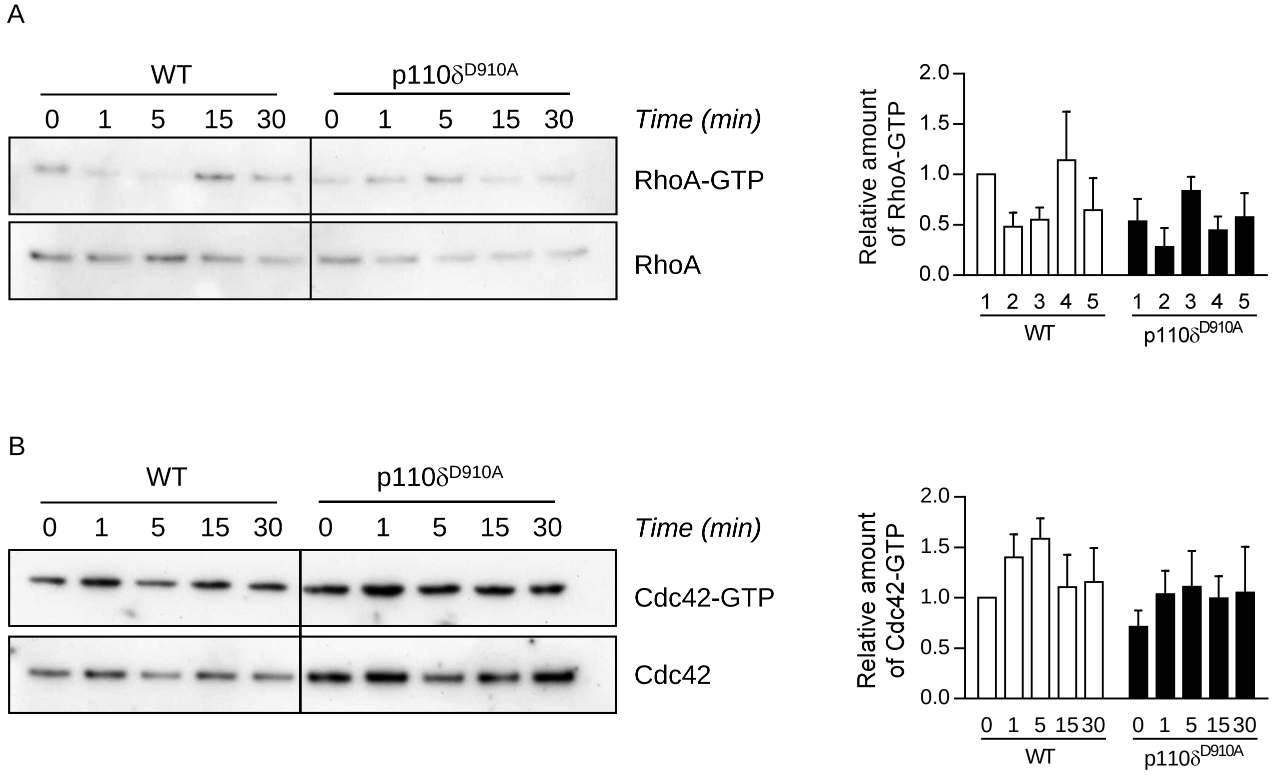

The active form of RhoA (A) or Cdc42 (B) was purified by GST pull-down from WT or p110δ^{D910A} T cell lysate after stimulation with an anti-CD3 mAb for the indicated times. For quantification, the relative amount of RhoA-GTP or Cdc42-GTP was normalized according to the total amount of the respective total protein. The data are reported as the fold intensity of the normalized GTP-bound small G protein observed at t=0 in the WT condition. Data are representative of three independent experiments.

Supplemental Figure 2

(to accompany main Figure 3)

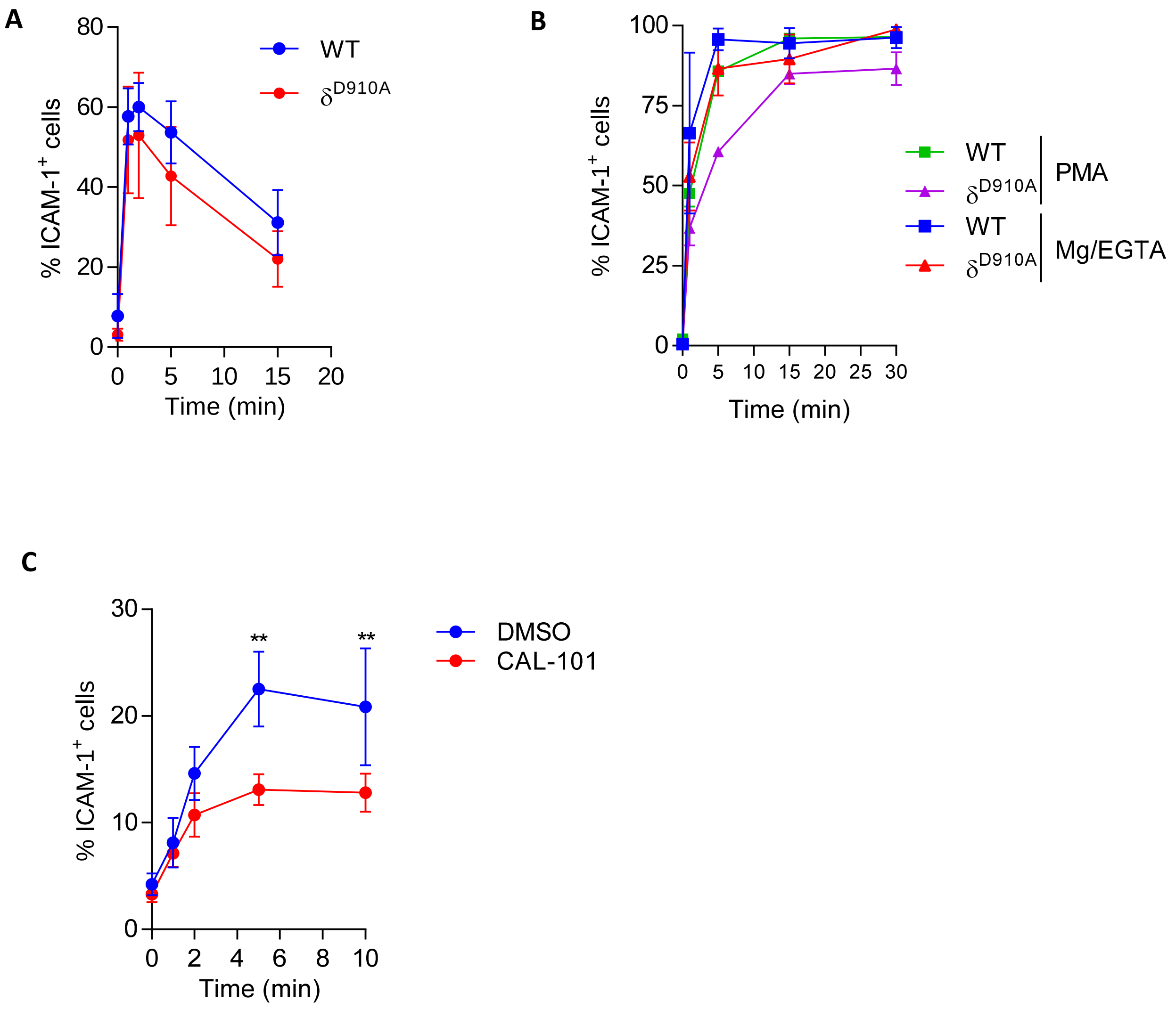

(A-B) CD4⁺ T cells were incubated with sc-ICAM-1-APC and stimulated with CCL19 (A), PMA or Mg/EDTA (B). After the indicated times, the reaction was stopped with 4% PFA. Bound scICMA-1-APC was detected by flow cytometry. Data show means of three (A) and two (B) independent experiments. (C) CD4⁺ T cells were incubated with DMSO or the p110δ-specific inhibitor CAL-101 (200 nM) before being stimulated with anti-CD3ε in presence of soluble ICAM-1 Fc. After fixation, bound ICAM-1 Fc was detected by staining with anti-human Fc-specific APC-labeled $F(ab')_2$ fragment. Data show means of three independent experiments.
